# Evaluating the effect of rice (*Oryza sativa* L.: SRNC05053-6-2) crude extract on psoriasis using in vitro and in vivo models

**DOI:** 10.1038/s41598-020-74634-4

**Published:** 2020-10-19

**Authors:** Sumate Ampawong, Kanchana Kengkoom, Passanesh Sukphopetch, Pornanong Aramwit, Watcharamat Muangkaew, Tapanee Kanjanapruthipong, Theerapong Buaban

**Affiliations:** 1 Department of Tropical Pathology, Faculty of Tropical Medicine, Mahidol University, Ratchawithi Road, Ratchathewi, Bangkok, 10400 Thailand; 2 Academic Service Division, National Laboratory Animal Center, Mahidol University, 999, Salaya, Puttamonthon, Nakorn Pathom, 73170 Thailand; 3 Department of Microbiology and Immunology, Faculty of Tropical Medicine, Mahidol University, Ratchawithi Road, Ratchathewi, Bangkok, 10400 Thailand; 4 Department of Pharmacy Practice, Faculty of Pharmaceutical Sciences and Center of Excellence in Bioactive Resources for Innovative Clinical Applications, Chulalongkorn University, PhayaThai Road, Phatumwan, Bangkok, 10330 Thailand; 5 The Academy of Science, The Royal Society of Thailand, Dusit, Bangkok, 10330 Thailand

**Keywords:** Experimental models of disease, Preclinical research

## Abstract

Psoriasis is mainly caused because of inappropriate immune responses in the epidermis. Rice (*Oryza sativa* L.: SRNC05053-6-2) consists of anthocyanin, which exhibits strong antioxidative and anti-inflammatory properties. This study aimed to evaluate the role of this black-coloured rice crude extract in alleviating the symptoms of psoriasis using human psoriatic artificial skin and an imiquimod-induced rat psoriasis model. Psoriasis-related genes, cytokines and chemokines were examined; in addition, the antioxidative and anti-inflammatory properties and the immunohistopathological features of this condition were studied. The results showed that the rice extract reduced the severity of psoriasis by (1) decreasing the epidermal thickness, acanthosis, hyperkeratosis, epidermal inflammation and degree of apoptosis induction via caspase-3, (2) increasing the expression levels of anti-inflammatory cytokines (IL-10 and TGF-β), (3) reducing the levels of pro-inflammatory cytokines (IL-6, IL-8, IL-20, IL-22 and TNF-α), chemokines (CCL-20) and anti-microbial peptides (psoriasin and β-defensin), (4) enhancing the antioxidative property (Nrf-2), (5) downregulating the levels of psoriasis-associated genes (*psoriasin*, *β-defensin*, *koebnerisin 15L* and *koebnerisin 15S*) and (6) upregulating the levels of psoriasis-improving genes (*caspase-14*, *involucrin* and *filaggrin*). Thus, the extract appears to exert therapeutic effects on psoriasis through its antioxidative and immunomodulatory properties.

## Introduction

Psoriasis, a noncontagious chronic skin condition characterised by the excessively rapid proliferation of keratinocytes, is a common T cell-mediated inflammatory skin disease; it is associated with autoimmune responses that mainly attack the target cells in the skin, particularly keratinocytes and melanocytes, leading to cellular hyperplasia and post-inflammatory hyperpigmentation, respectively^1–5^. Psoriasis is considered an incurable, long-term and inflammatory skin condition with periods of improvement and worsening. Furthermore, it is one of the most common skin conditions closely related to mental illness, particularly in patients who present with generalised lesions^6^. The pathogenesis of psoriasis mainly involves the imbalance of epidermal immune responses, which is divided into four stages: initialisation, innate immune response, adaptive immune response and amplification loop^7^. The initiation step is triggered by environmental and genetic factors (e.g. *LCE3B/LCE3C1*) and leads to epidermal alterations such an upregulation in the expression level of β-defensin, which results in the formation of self-DNA/RNA and LL37 complexes and the activation of interferon (INF)-γ. Subsequently, dendritic cells are stimulated into the maturation stage and play a pivotal role in mediating the innate immune response by secreting several cytokines, such as transforming growth factor (TGF)-β, tumour necrotic factor (TNF)-α and interleukin (IL)-6. Consequently, these cytokines initiate T cell-mediated adaptive immune responses through Th-17, Th-1 and Th-22 systems and lead to the increased production of IL-17A, IL-17F, IL-22, IL-21, TNF-α and INF-γ. Therefore, both Th-1 and Th-17 cytokines induce keratinocytes to produce IL-20, which increases the proliferation of the cells during the auto-amplification step. Moreover, IL-1β, IL-6 and TNF-α are produced by keratinocytes and contribute to the activation of dendritic cells, thereby promoting and increasing the severity of the epidermal inflammation.

The severity of psoriasis mainly depends on the degree of immune response in the epidermis. Hence, substances that reduce the degree of epidermal inflammation might serve as candidates for alleviating psoriasis in the skin. Researchers claimed that pigmented-rice comprises anthocyanin, which demonstrates strong antioxidative, anti-microbial and immunomodulatory properties^8,9^. The black-coloured rice *Oryza sativa* L.: SRNC05053-6-2 or Mali-Nil-Surin rice exhibits a dark purple pericarp and consists of high levels of anthocyanin, vitamins B-1 and -2, vitamin E and omega-3, -6 and -9; it is developed by several cooperation units in Thailand, particularly the (1) Surin Rice Research Center, (2) Sakon Nakhon Rice Research Center, (3) Division of Rice Research and Development, Rice Department, (4) Faculty of Technology and (5) Faculty of Science, Mahasarakham University, (6) Faculty of Medicine, Khon Kaen University and (7) School of Food Technology, Suranaree University^10^. Therefore, owing to its immunomodulatory and antioxidative properties, the crude extract of *O sativa* L.: SRNC05053-6-2 might demonstrate some beneficial effects on psoriasis of the skin. The present study aimed to evaluate the efficacy of *O sativa* L.: SRNC05053-6-2 extract in treating psoriasis using in vitro (full-thickness three-dimensional reconstituted human skin psoriasis) and in vivo (imiquimod-induced rat psoriasis) models. Several approaches, such as histopathology, immunohistochemistry, electron microscopy, Bio-Plex Multiplex immunoassay and quantitative reverse transcription-polymerase chain reaction (qRT-PCR), were used to determine the improvement criteria, which were based on the pathology and the levels of the cytokines, chemokines and genes related to psoriasis.

## Results

### A component of *O sativa* L.: SRNC05053-6-2 extraction

High-performance liquid chromatography (HPLC) indicated a high concentration of anthocyanin in the rice extract (Table 1), in addition to both vitamin E and C; however, vitamin D_2_ (ergocalciferol) was not detected in the extract. Furthermore, the extract comprised antioxidative properties when examined using the FRAP and DPPH antioxidative capacity assays.

**Table 1:** Composition of Nil Surin rice (*Oryza sativa* L.: SRNC05053-6-2) extract.

Component	Property	Test method
Anthocyanin	974.258 µg/50 mg/ml	HPLC
Vitamin E	1.06 mg α-tocopherol/100 g	In-house methods of Nutrition Institute, Mahidol University, Thailand
Vitamin D2	< 0.05 µg/100 g	AOAC (2016) 995.05
Ascorbic acid	3.35 mg/100 g	In-house methods of Nutrition Institute, Mahidol University, Thailand
Antioxidative activity	34,544.00 µmole TE/100 g	FRAP
	2,537.63 mgAA/100 g	DPPH

### Epidermal thickness and histopathological changes in psoriatic models after treatment with the extract

**Figure 1:**
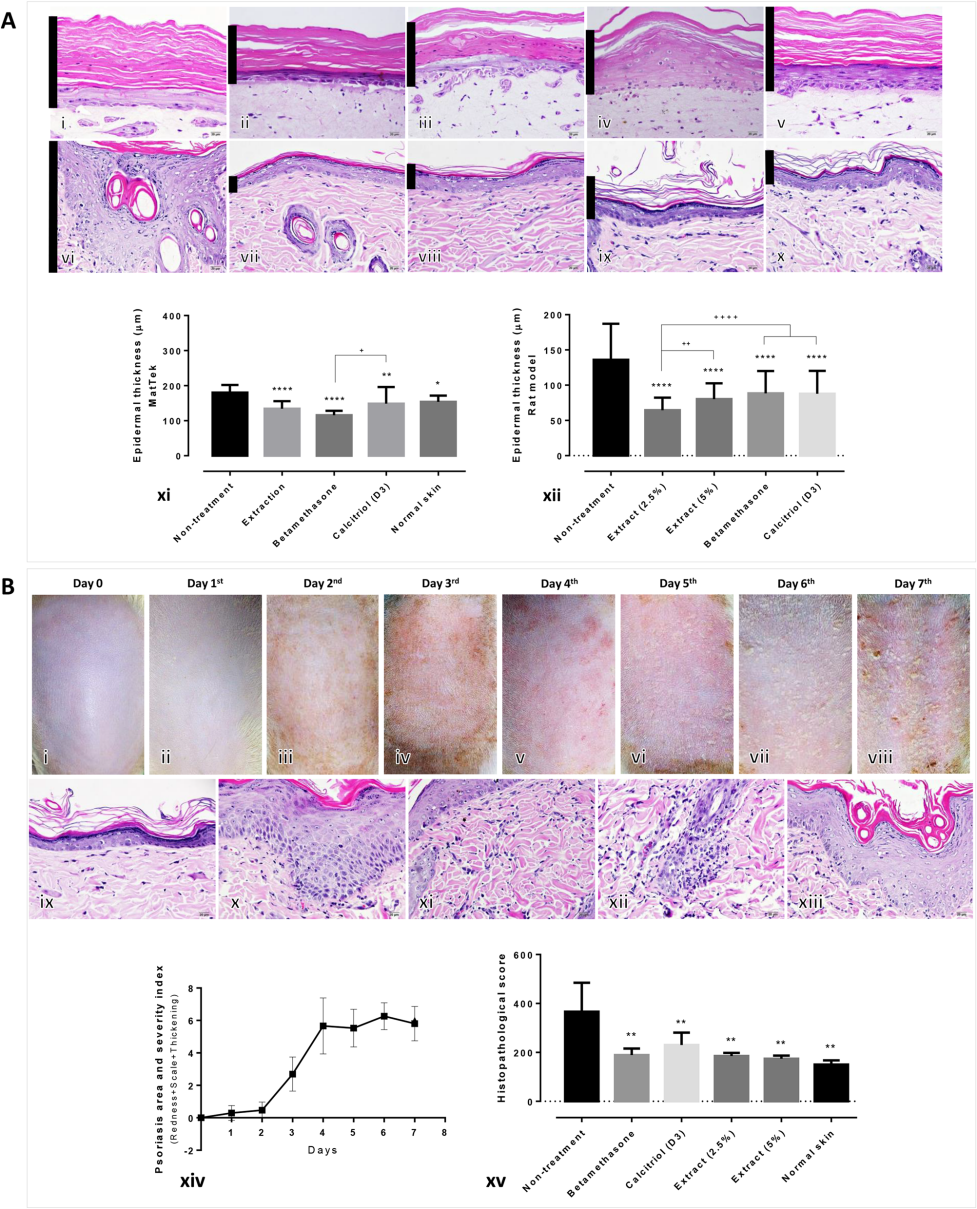
Histopathological evaluation of specimens from artificial psoriatic human skin and imiquimod-induced psoriatic rat skin: (**A**) Epidermal thickness at day 7 post-treatment in the artificial psoriatic (i–v) and rat psoriatic (xi, x) skins stained with hematoxylin and eosin (magnification, × 400). (i, vi) non-treatment; (ii, vii) extraction; (iii, viii) betamethasone; (iv, ix) calcitriol (D3); (v, x) normal skin groups. Bar graphs comparing the epidermal thickness in the artificial psoriatic (xi) and rat psoriatic (xii) skins. (**B**) Anatomical and histopathological appearances in imiquimod-induced psoriasis rats; gross skin lesion after induction with 62.5 mg of imiquimod from day 0 to day 7 (i–viii). Histopathological changes in rat psoriatic skin after 7 days of induction (ix–xiii). Ix, normal epidermis; x, acanthosis; xi, dermatitis; xii, folliculitis; xiii, hyperkeratosis. (xiv, xv) Line graph comparing the psoriatic area and severity index from day 0 to day 7 post-induction in each group (xiv). Bar graph comparing the histopathological scores among each treatment group (xv).

After treatment with the extract, the artificial psoriatic in vitro model demonstrated a significant decrease in epidermal thickness in all the treatment groups [extraction, betamethasone and calcitriol (D3)], when compared with the non-treated and intact skin specimens (Fig. 1A; i–v, xi). Similarly, a significant reduction in epidermal thickness was observed in all treatment groups in the imiquimod-induced psoriatic rat model when compared with the non-treated skin (Fig. 1A; vi–x, xii). Prior to the treatment, the imiquimod-induced psoriatic rats exhibited skin lesions such as redness, scaling and thickening (Fig. 1B). The psoriatic area and severity scores were rapidly increased 2 days post-induction and remained high on day 4 until the first day of treatment (Fig. 1B; xiv). The main epidermal histopathological changes observed were acanthosis with an increase in the number of rete ridges, dermatitis, folliculitis and hyperkeratosis (Fig. 1B; ix–xiii). Histopathological scores revealed that all treatment groups exhibited a significantly lower score than those in the non-treated rats (Fig. 1B; xv).

### Antiapoptotic, antioxidative and anti-inflammatory effects of the extract on the psoriatic skins

**Figure 2:**
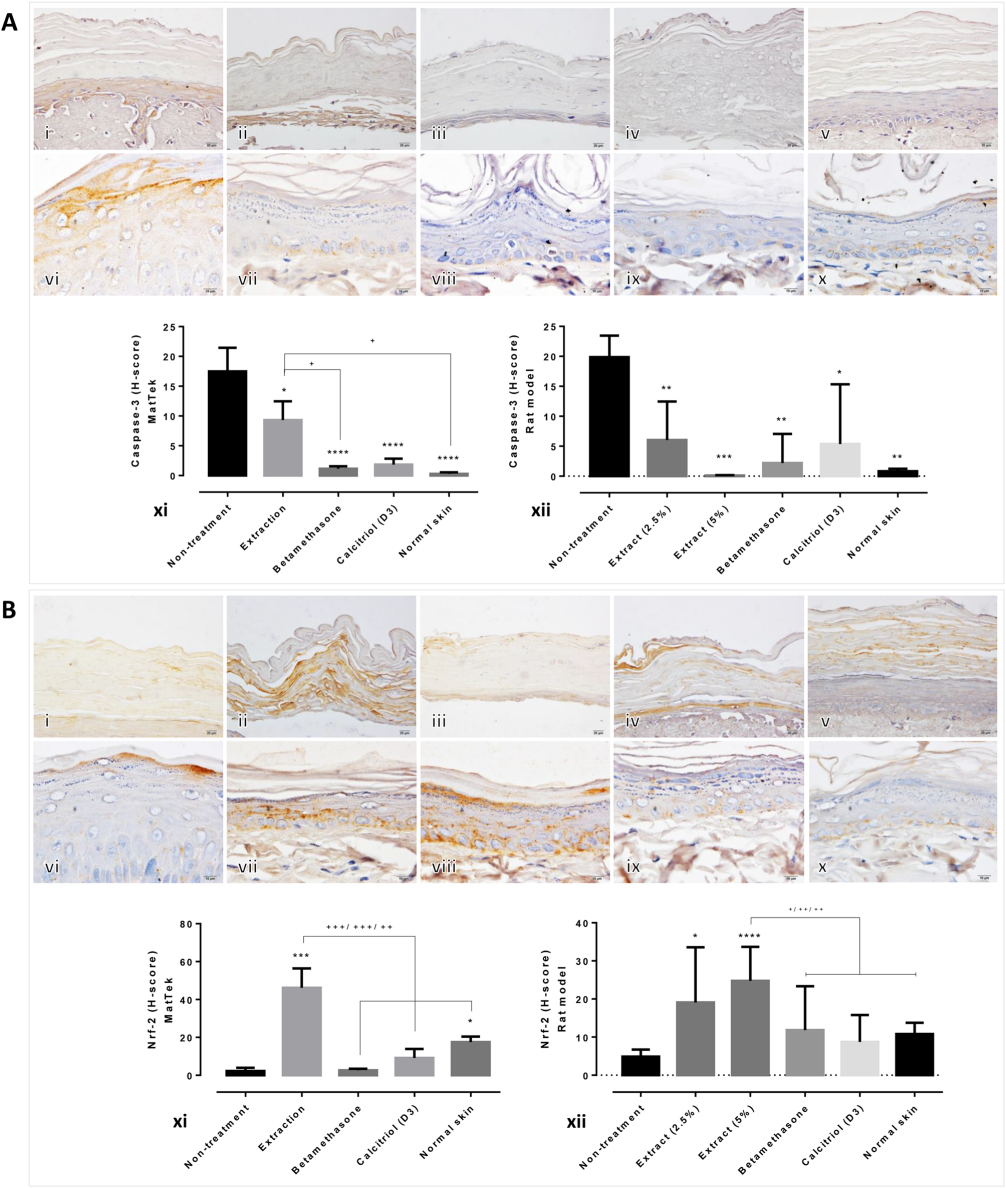
Epidermal apoptotic and antioxidative levels in artificial psoriatic human and imiquimod-induced psoriatic rat skins: (**A**) Caspase-3 immunolabeling of the epidermis at day 7 post-treatment in the artificial psoriatic (i–v) and rat psoriatic (vi–x) skins (magnification, × 400). (i, vi) non-treatment; (ii, vii) extraction; (iii, viii) betamethasone; (iv, ix) calcitriol (D3); and (v, x) normal skin. (xi, xii) Comparison of epidermal caspase-3 expression levels in artificial psoriatic (xi) and rat psoriatic (xii) skins. (**B**) Nrf-2 immunolabeling of the epidermis at day 7 post-treatment in the artificial psoriatic (i–v) and rat psoriatic (vi–x) skins (magnification, × 400). i and vi, non-treatment; (ii, vii) extraction; (iii, viii) betamethasone; (iv, ix) calcitriol (D3); and (v, x) normal skin. (xi, xii) Comparison of Nrf-2 epidermal expression in the artificial psoriatic (xi) and rat psoriatic (xii) skins.

Immunohistochemical staining of caspase-3, Nrf-2, IL-10, TGF-β, β-defensin and CCL-20 were performed to evaluate the antiapoptotic, antioxidative and anti-inflammatory properties of the extract on the psoriatic skin models with or without treatment. The results demonstrated that caspase-3 expression levels in all the treatment groups were significantly lower than those in non-treated skin, in both the in vitro and in vivo psoriatic models (Fig. 2A). However, the expression level of caspase-3 in the artificial psoriatic skin specimen, which received standard treatment, was significantly lower than that observed in the specimens that were treated with the extract. The antioxidative capacity, observed by determining the Nrf-2 expression level, was significantly higher in the groups treated with the extract, when compared with those in the other treatment groups in the artificial and rat psoriatic models (Fig. 2B).

**Figure 3:**
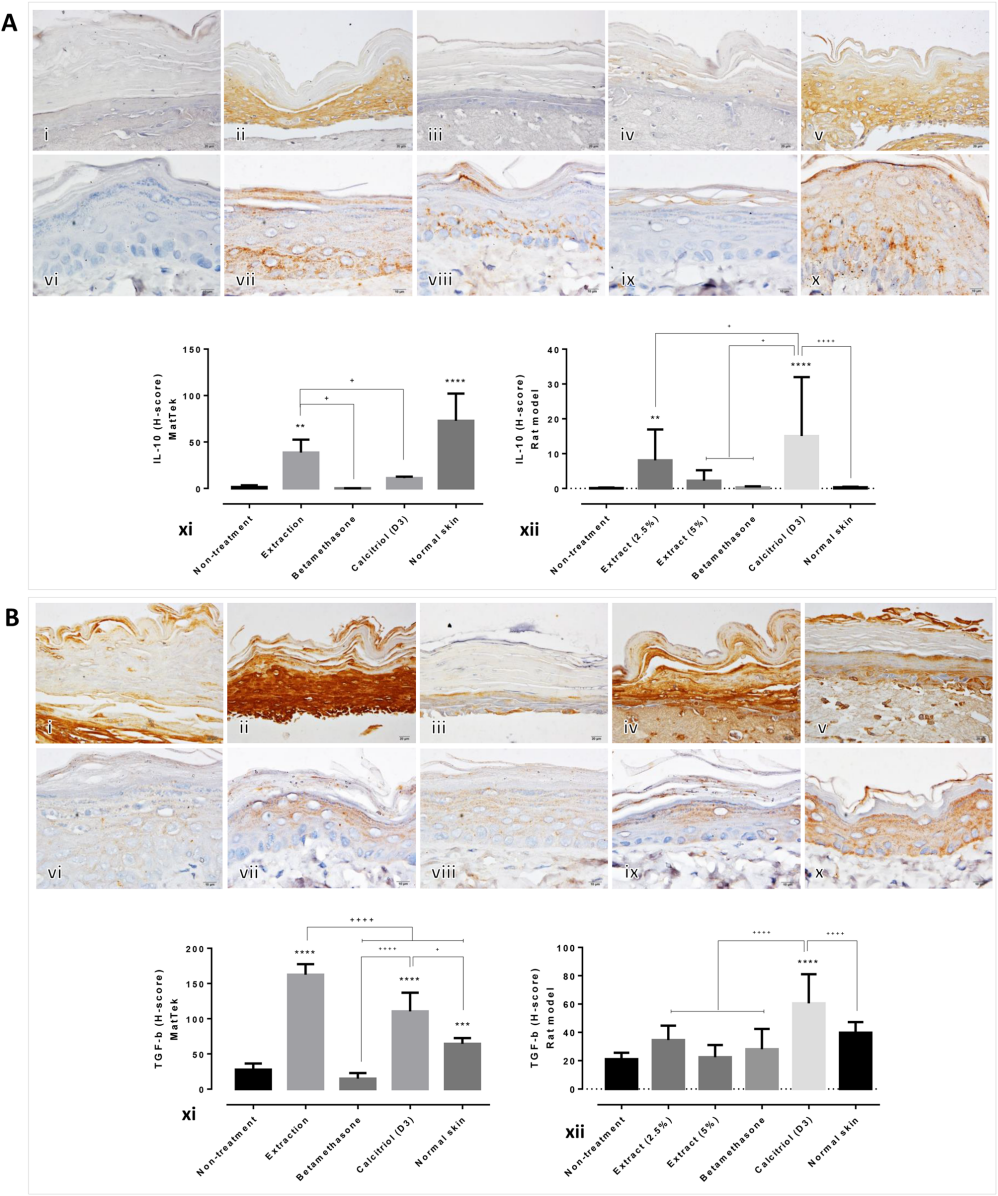
Epidermal anti-inflammatory cytokine levels in artificial psoriatic human and imiquimod-induced psoriasis rat skin specimens: (**A**) IL-10 immunolabeling of the epidermis at day 7 post-treatment in the artificial psoriatic (i–v) and rat psoriatic (vi–x) skins (magnification, × 400). (i, vi) non-treatment; (ii, vii) extraction; (iii, viii) betamethasone; (iv, ix), calcitriol (D3); and (v, x), normal skin. (xi, xii) Bar graph comparing the expression levels of epidermal IL-10 among the various groups in the artificial psoriatic (xi) and rat psoriatic (xii) skin specimens. (**B**) TGF-β immunolabeling of the epidermis at day 7 post-treatment in the artificial psoriatic (i–v) and rat psoriatic (vi–x) skins (magnification, × 400). (i, vi) non-treatment; (ii, vii) extraction; (iii, viii) betamethasone; (iv, ix), calcitriol (D3); and (v, x) normal skin. (xi, xii) Bar graph comparing the expression levels of epidermal TGF-β among the various groups in the artificial psoriatic (xi) and rat psoriatic (xii) skins.

Compared with the non-treated skin and standard treatment groups, IL-10 expression was found to be significantly increased in artificial skin treated with the extract; likewise, the expression levels of IL-10 in the rat psoriatic skins treated with 2.5% of the extract and calcitriol were significantly higher than those in the other groups (Fig. 3A). The expression levels of TGF-β in the artificial skin specimens treated with the rice extract and calcitriol were significantly higher than those in the other groups (Fig. 3B; i–v, xi). Moreover, only calcitriol-treated rat psoriatic skin demonstrated significantly upregulated levels of TGF-β compared with the other groups (Fig. 3B; vi–x, xii).

**Figure 4:**
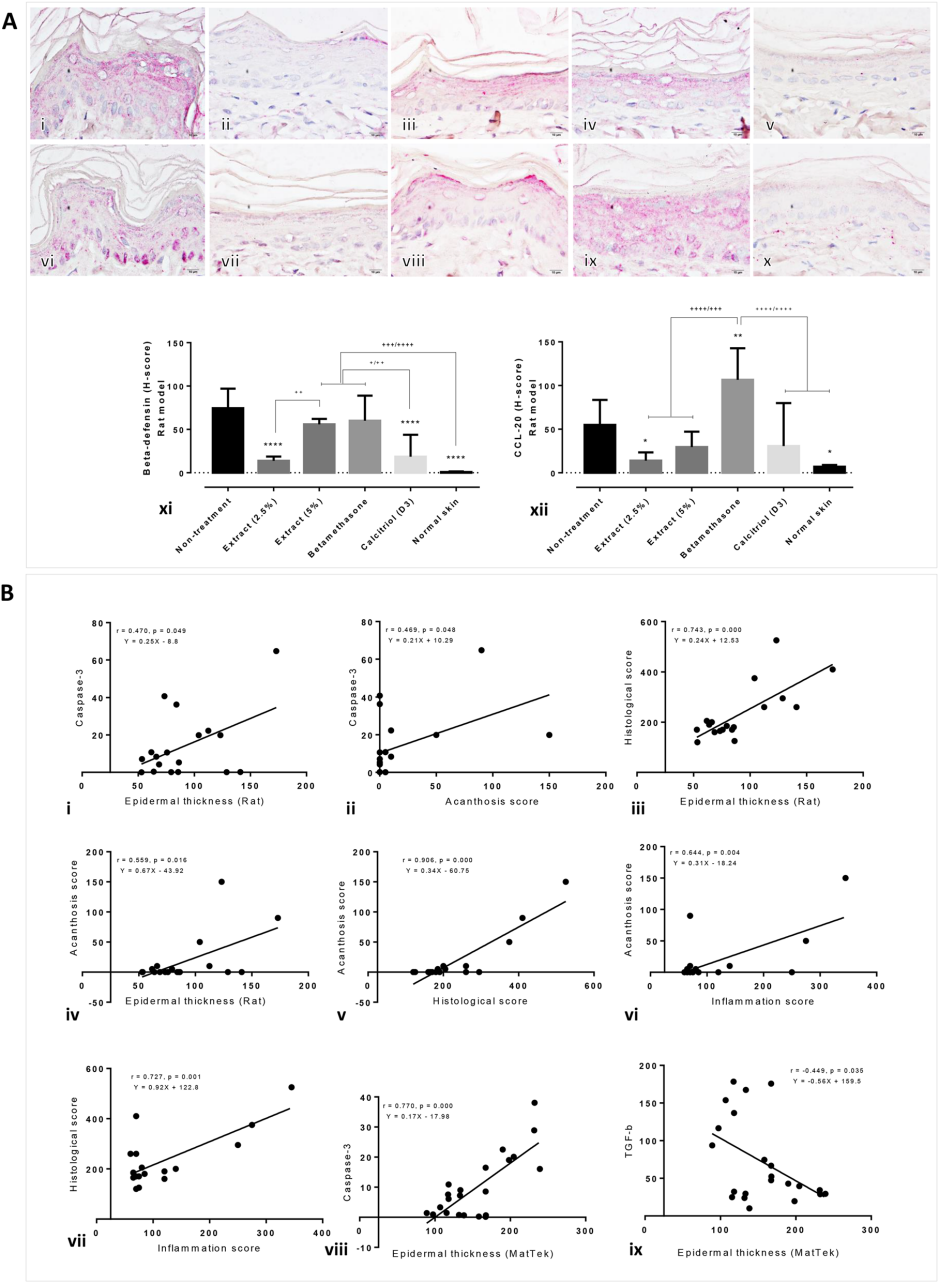
Epidermal psoriatic cytokine levels in artificial psoriatic human skin and imiquimod-induced psoriasis rat skin. (**A**) β-defensin (i–v) and CCL-20 (vi–x) immunolabeling of the rat psoriatic epidermis at day 7 post-treatment (magnification, × 400). (i, vi) non-treatment; (ii, vii) 2.5% extract; (iii, viii) 5% extract; (iv, ix) betamethasone; (v, x) calcitriol (D3). Bar graph comparing the expression levels of β-defensin and CCL-20 among the various groups in the artificial psoriatic (xi) and rat psoriatic (xii) skins. (**B**) Graphs (i–ix) showing correlations among the expression levels of the markers and the histopathological scores.

In addition, β-defensin expression levels were significantly decreased in rat psoriatic skin specimens treated with the extract (2.5%) and calcitriol, and the CCL-20 expression level was significantly downregulated in the 2.5% extract-treated rats, compared with those in the other treatment groups (Fig. 4A). The betamethasone-treated rats showed higher expression levels of CCL-20 than any of the other treatment groups. Spearman’s correlation test revealed that the expression level of epidermal caspase-3 was positively correlated with the epidermal thickness and acanthosis scores; alternatively, a negative correlation was observed between TGF-β and the epidermal thickness (Fig. 4B). Furthermore, the epidermal thickness was positively correlated to the acanthosis and histological scores. Additionally, positive correlations were observed between the (1) histological and inflammation scores, (2) histological and acanthosis scores and (3) acanthosis and inflammatory scores.

### Effect of rice extract on the expression levels of related psoriatic genes in artificial skin

**Figure 5:**
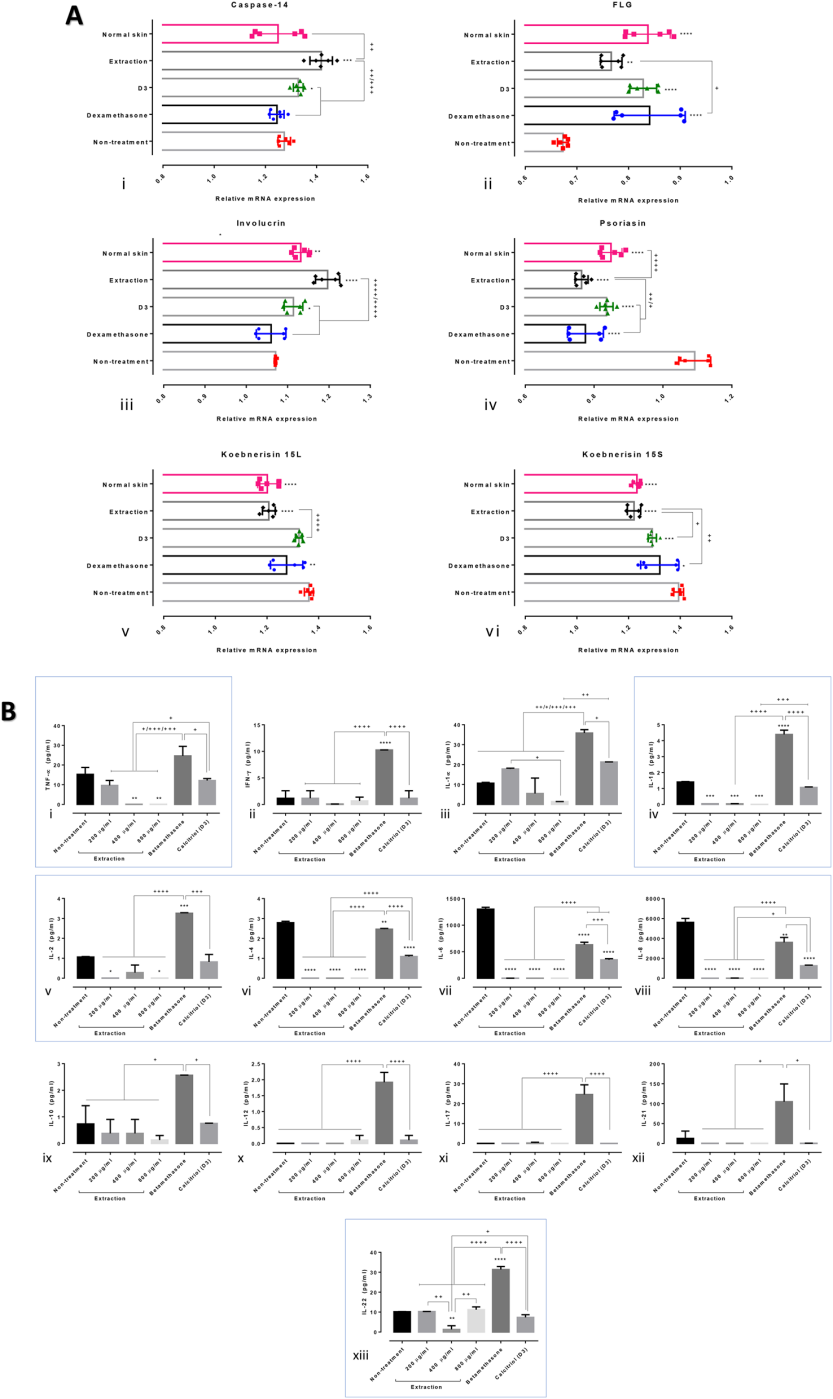
Gene expression and cytokine production levels in artificial psoriatic human skin. (**A**) Relative mRNA expression levels among the artificial psoriatic skin specimens with and without treatment. (i–vi) mRNA expression levels of caspase-14, filaggrin, involucrin, psoriasin, koebnerisin 15L and koebnerisin 15S, respectively. (**B**) Pro- and anti-inflammatory cytokines in the supernatants of the artificial psoriatic skins with or without treatment for 8 days. (i–xiii) Results of the Bio-Plex Multiplex immunoassay for tumour necrotic factor (TNF)-α, interferon (IFN)-γ, interleukin (IL)-1α, IL-1β, IL-2, IL-4, IL-6, IL-8, IL-10, IL-12, IL-17, IL-21 and IL-22.

The mRNA expression levels of caspase-14, filaggrin, involucrin, psoriasin and koebnerisin 15L/S were examined in the artificial skin specimens treated with or without the rice extract, calcitriol and dexamethasone using qRT-PCR. The results demonstrated that epidermal caspase-14 was upregulated in skin specimens treated with the extract and calcitriol compared with that in the non-treatment group (Fig. 5A). Furthermore, the expression level of caspase-14 in specimens treated with extract was significantly higher than that in the calcitriol-treated specimens. When compared with that in the non-treated group, the mRNA levels of filaggrin were significantly higher in all the treatment groups; in addition, a significant difference was observed between the extract-treated and dexamethasone-treated specimens. The expression level of epidermal involucrin was higher in the extract- and calcitriol-treated specimens than in the non-treated skin specimens and the extract group presented with the highest expression level among all groups. Psoriasin was significantly downregulated in all the treatment groups compared with the non-treated skin specimens. Likewise, the expression level of koebnerisin was decreased following all treatments, except for the 15L-subclass in calcitriol-treated skin specimens. Significant correlations between the expression levels of these genes and some of the immunohistochemical markers (caspase-3, Nrf-2, IL-10 and TGF-β) were observed (Table 2). High expression levels of caspase-14 were related with the upregulation of Nrf-2 and involucrin and downregulation of psoriasin. The expression level of involucrin was positively correlated with those of Nrf-2, IL-10 and TGF-β, and it was negatively correlated with those of koebnerisin and psoriasin. Negative correlations between the expression levels of koebnerisin and Nrf-2, IL-10 and TGF-β were noted. The decreased expression levels of caspase-3 and Nrf-2 were associated with the increase in the expression levels of filaggrin and psoriasin.

**Table 2:** Significant correlations between the gene expression levels and some immunohistochemical markers.

Genes/markers	Correlation coefficient (p-value)							
	Caspase-3	Nrf-2	IL-10	TGF-β	Involucrin	Koebnerisin15L	Koebnerisin15S	Psoriasin
Caspase-14		0.702 (0.000)			0.854 (0.000)			− 0.667 (0.001)
Involucrin		0.879 (0.000)	0.615 (0.002)	0.564 (0.006)		− 0.583 (0.004)	− 0.467 (0.028)	− 0.641 (0.001)
Koebnerisin15L		− 0.715 (0.000)	− 0.861 (0.000)	− 0.502 (0.017)			0.667 (0.001)	
Koebnerisin15S		− 0.730 (0.000)	− 0.640 (0.001)					0.542 (0.009)
FLG	− 0.592 (0.004)							
Psoriasin		− 0.633 (0.002)					0.542 (0.005)	

### Cytokine production in artificial skin following treatment with the extract

Some of the important pro- and anti-inflammatory cytokines and chemokines were selected to evaluate the differences in their expression levels using the Bio-Plex Multiplex immunoassay. TNF-α, IL-1β, IL-2, IL-4, IL-6, IL-8 and IL-22 levels were significantly decreased after treatment with the rice extract compared with those in the non-treated skin specimens (Fig. 5B; i, iv–viii, xiii). The expression levels of TNF-α in the 400 and 800 µg/ml extract-treated specimens were significantly lower than those in the 200 µg/ml extract-treated specimens and those that received standard treatments. IL-1β, IL-2, IL-4, IL-6 and IL-8 levels were significantly lower than those in the specimens that received standard treatment. In addition, the expression level of IL-22 was the lowest following treatment with 400 µg/ml of the extract compared with those in the other groups. Conversely, INF-γ, IL-1α, IL-10, IL-12, IL-17 and IL-21 levels were not different between the rice extract-treated and -untreated specimens. However, the specimens treated with betamethasone demonstrated significantly higher levels of TNF-α, IFN-γ, IL-1α, IL-1β, IL-2, IL-4, IL-6, IL-8, IL-10, IL-12, IL-17, IL-21 and IL-22 than the other specimens.

### Antimelanogenicity and immunomodulatory capacities of the rice extract

**Figure 6:**
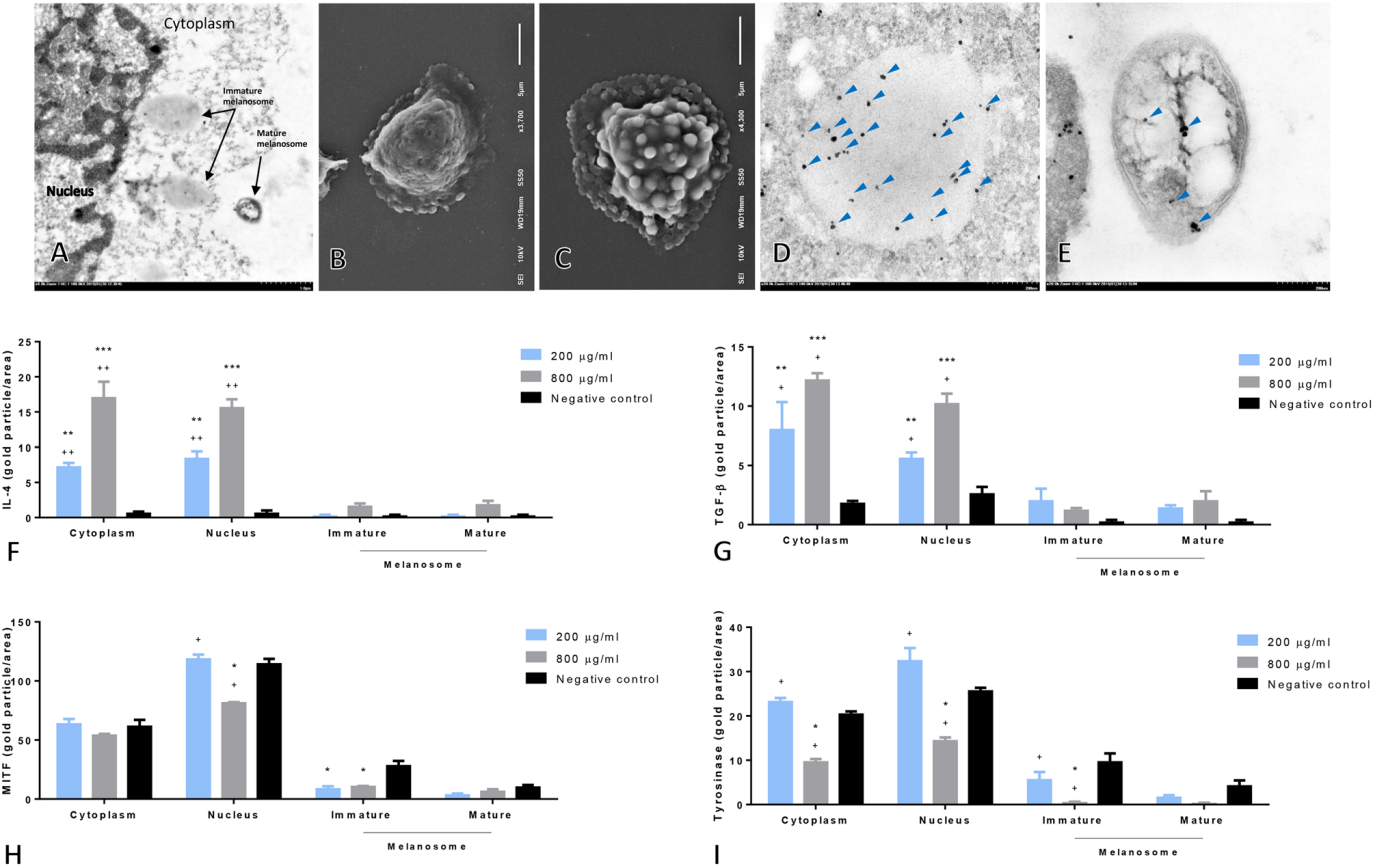
Antityrosinase, anti-melanocytic and anti-allergic properties of rice extract on melanocytes. Transmission electron image of allergic melanocyte (**A**) and scanning electron images of allergic melanocyte with (**B**) or without treatment using rice extract (**C**). Immunogold labelling of MITF in immature (**D**) and mature melanocytes (**E**). Bar graph comparisons of IL-4, TGF-β, MITF and tyrosinase expression levels in melanocytes among the various groups (**F**–**I**).

Electron microscopy was used to examine the antimelanogenic and immunomodulatory capacities of the extract on PEG-induced allergic melanocytes. Gold immunolocalisations of IL-4, TGF-β, microphthalmia-associated transcription factor (MITF) and tyrosinase were found in the cytoplasm and nucleus of the melanocytes and mature and immature melanosomes (Fig. 6A). Scanning electron micrographs indicated that several melanosomes were produced in allergic melanocytes that were not treated with the extract compared with those in the extract-treated cells (Fig. 6B, C). MITF immunoreactivity was observed in the transmission electron micrographs in both immature and mature melanosomes (Fig. 6D, E). The expression levels of IL-4 and TGF-β in melanocytes treated with 200 and 800 µg/ml of rice extract were significantly higher than those in the non-treated cells, particularly in the nucleus and cytoplasm (Fig. 6F, G). The levels of MITF and tyrosinase in immature melanocytes treated with 200 and 800 µg/ml of the rice extract were lower than those in the non-treated cells (Fig. 6H, I). In addition, MITF and tyrosinase levels were downregulated in melanocytes treated with 800 µg/ml of the extract compared with those in melanocytes treated with 200 µg/ml of the extract and the non-treated cells.

## Discussion

Several natural products, such as curcumin^11,12^, cannabinoid^13^, anthocyanin (and its derivatives), were developed for the alleviation of psoriasis^14–19^. These products exert the desired effects on psoriasis via different mechanisms. Curcumin extract, when applied both topically and orally, acts as an antiseptic and decreases inflammation by downregulating the levels of inflammatory cytokines such as TNF-α, IFN-γ, IL-2, IL-17A, IL-17F, IL-22 and IL-23, thereby reducing both parakeratosis and hyperkeratosis in the psoriatic lesions^11,12^. Moreover, curcumin is easily available and inexpensive, and it is known to exhibit low oral bioavailability; however, new delivery system formulations and clinical trials are being conducted to develop appropriate forms of this product. Cannabinoid extract containing tetrahydrocannabinol (THC) and cannabidiol (CBD) is used for the treatment of psoriasis. Both THC-mediated inhibition of keratinocyte proliferation and cannabinol (CB)-1 activation are believed to be crucial for the treatment of psoriasis via the reduction of inducible factor-1 α (HIF-1 α), vascular endothelial growth factor, basic fibroblast growth factor (bFGF), angiopoietin-2, IL-8, IL-17 and IL-2^13^. Anthocyanins demonstrate potent antioxidative, anti-inflammatory and anti-proliferative properties and are obtained from natural pigmented fruits, rice and vegetables^20^. Anthocyanin and its derivatives decrease psoriasis by inhibiting key kinases and cytokines^15^, reducing keratinocyte proliferation^16,17^ and controlling the expression of late cornified envelope genes^14,21^.

New candidates for the treatment of psoriasis are being investigated. In the current study, the effect of crude extract from *Oryza sativa* L.: SRNC05053-6-2 consisting of high levels of anthocyanin were evaluated. The results from in vitro and in vivo studies indicated that the extract exhibited some beneficial effects on psoriasis, as demonstrated by the reduction in the epidermal pathologies (Fig. 1A, B). Epidermal thickening, acanthosis, hyperkeratosis and inflammation were reduced along with a decrease in the degree of cellular apoptosis (downregulation of caspase-3; Fig. 2A). The antioxidative environment as enhanced (upregulation of Nrf-2; Fig. 2B); furthermore, the expression levels of anti-inflammatory cytokines were increased (upregulations of IL-10 and TGF-β; Fig. 3) and those of some chemokines were decreased (downregulations of β-defensin and CCL-20; Fig. 4A). qRT-PCR indicated that compared with the standard treatments, the extract could maintain the homeostasis as well as the functional and structural integrities of the epidermis by upregulating the mRNA expression levels of caspase-14, involucrin and filaggrin, thereby improving the skin conditions (Fig. 5A). The depletion of anti-microbial peptides such as psoriasin and koebnerisin (15L and 15S), which are mainly found in inflamed psoriatic skin, leads to epidermal degeneration and necrosis^22^ and enhances the curable effect of the extract on psoriatic skin conditions. In the present study, the reduction in epidermal apoptosis and improvement in epidermal integrity were closely related to the antioxidative property of the extract via Nrf-2 expression. This finding is consistent with that reported by Lee et al.^23^, wherein psoriasis vulgaris was found to be attenuated in human skin following a reduction in the redox burden and the subsequent oxidative damage to normal keratinocytes through the activation of the Nrf-2 pathway. In the present study, epidermal thickening was positively correlated with the expression levels of caspase-3, an apoptotic inducer, and negatively correlated with that of TGF-β, a multipotent cytokine that regulates both cell growth and differentiation. Owing to its anti-inflammatory and anti-keratinocyte proliferative activities, the downregulation of TGF-β signalling was reported in human psoriatic conditions^24^. In addition, the extract demonstrated antimelanogenic properties through its anti-inflammatory and antityrosinase activities, as characterised by the upregulation of IL-4 and TGF-β and downregulation of MITF and tyrosinase, respectively, in allergic melanocytes (Fig. 6), thus leading to a reduction in melanosome production (Fig. 6A–E). Owing to its antityrosinase property, anthocyanin extract can inhibit melanogenesis in the melanocyte^25,26^. This effect of the extract on melanin biogenesis is very important for improving psoriatic skin conditions during treatment with PUVA or photochemotherapy, which can lead to sub-epidermal melanin-containing phagocytosis^27^. Furthermore, researchers well recognised that melanocytes act as new co-operated target cells with keratinocytes in provoking autoantigens and autoimmunity in psoriasis^2^. Therefore, the beneficial effect of the extract on melanogenesis inhibition is desired for the development of anti-psoriactic agents, in addition to the anti-inflammatory and anti-epidermal thickening effects.

Several psoriasis-related cytokines and chemokines are considered important factors in the pathogenesis of psoriasis as mentioned above. In the present study, Bio-Plex Multiplex immunoassay revealed that the rice extract reduced inflammatory responses by (1) regulating the innate immune response via enhanced expression levels of TGF-β and IL-10, (2) modulating the adaptive immune response through the downregulation of IL-6, IL-8, IL-22 and TNF-α and (3) attenuating the auto-amplification loop via the reduction of IL-20, CCL-20 and β-defensin. Cumulatively, these findings demonstrate that the beneficial effects of the extract in ameliorating the severity of psoriasis.

In summary, the present study explored the antipsoriatic property of the crude extract from *Oryza sativa* L.: SRNC05053-6-2 or Mali-Nil-Surin rice using in vitro and in vivo models. The extract improved epidermal integrity by maintaining the expression levels of psoriasin, β-defensin, koebnerisin 15L, koebnerisin 15S, caspase-14, involucrin and filaggrin. In addition, inflammation had decreased owing to the antioxidative effects exerted via Nrf-2 and the immunomodulatory effects exerted by the regulation of IL-10, TGF-β, IL-6, IL-8, IL-20, IL-22, TNF-α and CCL-20 levels. Therefore, *O sativa* L.: SRNC05053-6-2 extract appears to be a potential candidate for the treatment of psoriasis.

## Methods

### Ethics statement

The animal experiments were performed with the permission of the National Laboratory Animal Center-ACUC, Mahidol University (approval number RA2019-21). The Thai Animals for Scientific Purposes Act, B.E. 2558, and the Guidelines for the use of animals of the National Research Council of Thailand were implemented. Sprague–Dawley rats were obtained from the National Laboratory Animal Center at Mahidol University. They were housed in a temperature-, ventilation- and humidity-controlled room under 12-h/12-h light/dark cycle conditions and fed with a standard diet ad libitum.

### Extract preparation

Mali-Nil-Surin rice (*Oryza sativa* L.: SRNC05053-6-2) was obtained from organic farms certified by the Ministry of Agricultural and Cooperative, Thailand. The pericarp was milled and rice extract was prepared via ethanolic extraction by the Thai-China Flavours and Fragrances Industry Co., Ltd. (TCFF) using a hi-speed herb extractor (HX-50, UK). The main active ingredients of the extract (anthocyanin, ascorbic acid and α-tocopherol) and its antioxidative properties [total antioxidative activity; ferric reducing antioxidative power (FRAP) and 1,1-diphenyl-2-picrylhydrazyl (DPPH) assays] were evaluated by TCFF and the Nutrition Institute, Mahidol University, Thailand.

### In vitro and in vivo effects of the extract on psoriasis

#### In vitro psoriasis model

Full-thickness three-dimensional reconstituted human skin models of psoriasis (PSE) and normal skin (NSE; MatTek, USA) were used to evaluate the ability of the extract to alleviate psoriasis. The PSE consisted of human psoriatic keratinocytes, highly differentiated epidermis and collagen-contracted fibroblasts, whereas the NSE consisted of intact human keratinocytes and other cells. A full-thickness artificial skin specimen was equilibrated at 37 °C and 5% CO_2_ for 24 h when received and maintained in SOR-300-FT-MM media throughout the experiment. Pre-warmed media with or without treatment (200 µg/ml extract, 1 µg/g calcitriol with 0.01 µg/g betamethasone [Xamiol] and 0.4 mg/ml dexamethasone) was added to the artificial skin and changed every 2 days until 8 days post-treatment. The experiments were performed in triplicates. The supernatant was collected at − 80 °C to determine the concentrations of the cytokines. The artificial skin was divided into two parts: one was fixed in 10% neutral buffer formalin (NBF) for histopathological examination, and the other was frozen at − 80 °C to evaluate the expression levels of psoriasis-related genes using qRT-PCR.

#### Peptidoglycan (PEG) induced melanocyte allergy

Recently, researchers showed that in addition to keratinocytes, melanocytes contribute to the severity of psoriasis by targeting the human leukocyte antigen class I allele, the main psoriasis risk gene^2^. To demonstrate the anti-inflammatory and antityrosinase activities of the extract, a melanocyte allergic model was created via PEG-induced inflammation^28^. The human melanoma cell line (ATCC CRL-1676) was cultured in Eagle’s Minimum Essential Medium supplemented with 10% (v/v) heat-inactivated fetal bovine serum and 1% (v/v) penicillin/streptomycin. The cells were cultured (density, 10^6^ cells/ml) and maintained at 37 °C with 5% CO_2_ in a humidified incubator. The melanocytes were co-cultured with 10 µg/ml of *S. aureus* PEG (Sigma-Aldrich, USA) for 24 h. After induction, the cells were treated with or without 200 and 800 µg/ml of the rice extract for 24 h. After the cells were trypsinised with 0.25% Trypsin-EDTA and washed in sucrose phosphate buffer (SPB), they were centrifuged at 3500 rpm (4 °C) for 5 min. The melanocyte pellets were collected and fixed in 2.5% glutaraldehyde and SPB for 1 h at room temperature.

#### Induced psoriasis rat model

To induce psoriasis in the rat model, 62.5 mg of imiquimod was topically applied to the dorsal shaved rat skin (limited to 3 × 3 cm^2^ area) daily for 7 days^29^. Thirty rats were equally divided into six groups (5 rats/group) based on the following different test materials used for 7 days: cream base (non-treatment group); 2.5%, 5% and 10% of rice extract; 3 µg/ml of calcitriol ointment; and 0.1% betamethasone. After the induction period, imiquimod was continually applied to the rats to maintain the psoriatic skin lesion. All test materials were applied 2 times/ day (b.i.d.) after the application of imiquimod for 1 h. The skin lesion was grossly scored every day by focusing on the erythema, scale and thickening using four grades: 0 = absent, 1 = mild, 2 = moderate and 3 = severe. At the end of the study, all rats were humanely euthanised by an overdose of carbon-dioxide inhalation. The skin lesions were collected and preserved in 10% NBF for histopathological examination.

### qRT-PCR

RNA from artificial skin was isolated using TRIzol Reagent (Invitrogen, USA), according to the manufacturer’s instructions. Complementary DNA synthesis and amplification were performed using the CFX96 Touch Real-time PCR Detection system thermocycler and KAPA SYBR FAST One-Step qRT-PCR Kit (KAPABIOSYSTEMS, USA), according to the manufacturers’ protocols. The primer pairs used are presented in Table 3. The level of mRNA was normalised to the levels of β-actin or TGase-1 mRNA. Subsequently, the relative mRNA expression levels were calculated using the 2^−ΔΔCt^ method.

**Table 3:** Primers used for qRT-PCR analysis.

Gene	Forward	Reverse
FLG	5′-AAGGTTCACATTTATTGCCAAA-3′	5′-GGATTTGCCGAAATTCCTTT-3′
β-Actin	5′-TGACGGGGTCACCCACACTGTGCCCATCTA-3′	5′-CTAGAAGCATTTGCGGTGGACGATGGAGGG-3′
Caspase-14	5′-AAATGAGCAATCCGCGGTCTTTGG-3′	5′-CCGTGGAATAAACGTGCAAGGCAT-3′
Involucrin	5′-CTCCACCAAAGCCTCTGC-3′	5′-CTGCTTAAGCTGCT GCTC-3′
TGase-1	5′-TGAATAGTGACAAGGTGTACTGGCA-3′	5′-GTGGCCTGAGACATTGAGCAGCAT-3′
psoriasin-hS100A7	5′-AGACGTGATGACAAGATTGAC-3′	5′-TGTCCTTTTTCTCAAAGACGTC-3′
Koebnerisin 15S-hS100A15S	5′-CAAGTTCCTTCTGCTCCATCTTAG-3′	5′-AGCCTTCAGGAAATAAAGACAATC-3′
Koebnerisin 15L-hS100A15L	5′-ACGTCACTCCTGTCTCTCTTTGCT-3′	5′-TGATGAATCAACCCATTTCCTGGG-3′

### Bio-plex multiplex immunoassay

To determine the cytokines involved in the innate and adaptive immune responses and the auto-amplification loop immunity, which are closely related to the pathogenesis of psoriasis, the Bio-Plex Multiplex immunoassay (BioRad, USA) was performed. Luminex magnetic beads were used to quantify some of the following cytokines: tumour necrotic factor (TNF)-α, interferon (IFN)-γ, interleukin (IL)-1α, IL-1β, IL-2, IL-4, IL-6, IL-8, IL-10, IL-12, IL-17, IL-21 and IL-22. After harvesting the cells from the artificial skin following treatment for 7 days, the supernatant was thawed and centrifuged at 1000*g* for 10 min. The negative and positive controls were supplied in the kit.

### Histopathological examination

To assess the severity of the histopathological changes in the artificial and rat psoriatic skins, standard tissue processing consisting of dehydration, infiltration, embedding, sectioning and staining with hematoxylin and eosin was performed. The histopathological score was ascertained following examination under a light microscope. The epidermal thicknesses of the artificial and rat psoriatic skin specimens were measured using an imaging analysis programme (ImageJ Version 1.36; National Institutes of Health; Bethesda, MD, USA). The histopathological changes in rat psoriatic skin were determined by observing the presence of acanthosis and elongation of rete ridges, the thickness of the epidermis and the presence of hyperkeratosis, folliculitis and dermatitis; the changes were scored using the H-score, as described previously^30–39^. The H-score (0–300) was calculated by multiplying the severity score (0–3; 0 = absent, 1 = mild, 2 = moderate and 3 = severe) with the extent of distribution (0–100%).

### Immunohistochemical studies

To demonstrate the resolutive, antioxidative and immunomodulatory properties of the extract on both artificial and rat psoriasis skin specimens, the immunostaining, as described in our recent studies^30–35,38,39^, of caspase-3, nuclear factor erythroid 2-related factor (Nrf)-2, interleukin (IL)-10, TGF-β, β-defensin and CCL-20 were performed using rabbit isotype polyclonal antibodies (MyBioSource, USA). After deparaffinisation and dehydration, the antigenicity of the section was enhanced using heated citrate buffer (pH 6.0). The blocking steps were performed using peroxidase, and non-specific binding was blocked using EnVision FLEX/ horseradish peroxidase (HRP) blocking reagent (DAKO, Denmark, K8002). The sections were incubated with the primary antibody for 1 h and labelled with polymer HRP anti-mouse/rabbit (DAKO) for 20 min, followed by visualisation with diaminobenzidine (DAB; DAKO). Finally, the sections were counterstained with hematoxylin and mounted with DEPEX (Electron Microscopy Sciences, USA). The immunolabeling of all the markers of interest was semi-quantified based on the H-score (percentage of expression × intensity score) using the image analysis programme to localise an area of expression. Briefly, psoriatic full-thickness skin lesions were captured as colour images (at least five images/ animal). The immunolabeled area was located using the threshold mode, and the images were converted to grayscale. Finally, the area of expression (percentage) was measured. The intensity score was graded as follows: 0 = negative staining; 1 = low-intensity staining; 2 = moderate-intensity staining; and 3 = high-intensity staining.

### Electron microscopy

#### Transmission electron microscopy

All the fixed melanocyte pellets were fixed with 1% osmium tetroxide in 0.1 M SPB for 1 h at room temperature. Next, the specimens were dehydrated in graded ethanol, infiltrated with LR White resin (EMS, USA), embedded in capsule beams and finally polymerised at 65 °C for 48 h. All specimens were cut into 90–100-nm thick section and placed on a nickel grid for immunogold staining.

#### Scanning electron microscopy

Glutaraldehyde-fixed melanocytes were washed with 0.1 M SPB and secondarily fixed with 1% osmium tetroxide in 0.1 M SPB for 1 h at room temperature. The pellets were dehydrated with graded ethyl-alcohol, smeared on a glass coverslip, dried in a desiccator and coated with gold film up to a thickness of 20 nm using a sputter coater (K550; EMITECH, UK). All specimens were examined under a scanning electron microscope (JSM-6610LV; JEOL, Japan) with 10-kV acceleration voltage to determine the density of the melanosome in the melanocyte.

#### Immunogold labelling

To examine the anti-inflammatory and antimelanogenic capacities of the extract on PEG-induced melanocyte inflammation, immunogold electron microscopy was performed, as described in our recent studies^32–34^, using rabbit anti-IL-4, TGF-β, MITF and tyrosinase (MyBioSource, USA). Sections were blocked using 50 mM glycine and 5% bovine serum albumin (BSA; EMS, USA). The sections were incubated with primary antibodies for 1 h and then with immunoglobulin (Ig)G conjugated with 10 nm gold particles (EMS, USA) for 1 h. A silver enhancement kit (Aurion R-Gent SE-EM kit, EMS, USA) was used after rigorously washing the sections with distilled water. Finally, the sections were stained with lead citrate and uranyl acetate and examined under a transmission electron microscope (HT7700, HITACHI, Japan). The number of gold particles labelled in the nucleus and cytoplasm of the mature and immature melanosome was counted (in at least 50 cells/group).

### Statistics

Statistical analysis was performed using GraphPad Prism version 5, and data are expressed as mean ± standard error of the mean. Statistical significance was denoted as *p* < 0.05 (*, **, ***, or **** referred to a significant difference to non-treated group at *p*-value < 0.05, 0.01, 0.001, or 0.0001, respectively: +, ++, +++ or ++++ referred to a significant difference between match pair at *p*-value < 0.05, 0.01, 0.001 or 0.0001, respectively). The Kolmogorov–Smirnov test was used to determine the data distribution, and appropriate parametric and non-parametric *t*-tests or analysis of variance were chosen to compare the differences in histopathological, ultrastructural and immunohistochemical changes among groups. Spearman’s correlation test was used to determine the correlations in this study.

### Data availability

The data sets used and/or analysed during the current study are available from the corresponding author upon request.
